# Extra vascular lung water but not lung ultrasound predicts grade 3 pulmonary graft dysfunction and utilization of rescue therapies for severe hypoxemia after lung transplantation

**DOI:** 10.1186/2197-425X-3-S1-A233

**Published:** 2015-10-01

**Authors:** C Guervilly, S Lehingue, S Dizier, L Zieleskiewics, S Hraiech, XB D'journo, G Thomas, F Klazen, M Adda, A Roch, JM Forel, P Thomas, M Leone, L Papazian

**Affiliations:** Respiratory Distress and Severe Infection Critical Care Unit, North Hospital, Assistance Publique-Hôpitaux de Marseille, Marseille, France; Department of Anesthesiology and Critical Care Medicine, North Hospital, Assistance Publique-Hôpitaux de Marseille, Marseille, France; Department of Thoracic Surgery and Diseases of the Esophagus, Aix-Marseille University, North Hospital, Assistance Publique-Hôpitaux de Marseille, Marseille, France

## Intr

Primary graft dysfunction (PGD) is the result of pulmonary edema following lung transplantation. The definition [1] is based on Pa02/Fi02 and the presence of lung infiltrates on chest X-ray. Lung ultrasonography (LUS) and the extravascular lung water index (EVLWi) are reliable methods for quantification of lung edema [2, 3].

## Objectives

We tested if LUS and EVLWi were associated with grade 3 PGD.

## Methods

We prospectively included patients who underwent lung transplantation in one university teaching hospital over a 14-month period. LUS scores and EVLWi were assessed at ICU admission and during the following 48 hours period. We compared patients with grade 3 PGD with the others.

## Results

36 patients were included. Among them, 13 (36 %) had grade 3 PGD. EVLWi was significantly higher in the grade 3 PGD group at ICU admission, day one and at day 2.

A cut-off value of 14 ml/kg of EVLWi at admission predicts the progression to grade 3 PGD with a sensitivity of 82% and a specificity of 77% and was significantly associated with the need for rescue therapy for severe hypoxemia.

Less than a half of patients had all their LUS windows available. LUS scores did not discriminate patients with grade 3 PGD from the others.

## Conclusions

Contrary to LUS scores, EVLWi is a promising tool for early assessment of grade 3 PGD after lung transplantation.
